# Covid-19 as an opportunity to teach epistemic insight: findings from exploratory workshops on Covid-19 and science with students aged 15–17 in England

**DOI:** 10.1007/s43545-021-00243-1

**Published:** 2021-09-22

**Authors:** Berry Billingsley, Joshua M. Heyes, Mehdi Nassaji

**Affiliations:** Faculty of Arts, Humanities and Education, Canterbury Christ Church University, North Holmes Road, Canterbury, CT1 1QU UK

**Keywords:** Science education, Covid-19, Epistemic insight, Misinformation, Multi-disciplinarity

## Abstract

The contributions of science and scientists to combatting Covid-19 have been at the forefront of media attention throughout 2020 and early 2021, exposing the public to the processes of science in an unprecedented manner. The pandemic has highlighted the necessity of scientists working collaboratively with other disciplines in informing thinking about a complex, evolving real-world problem. This draws attention to recent efforts, both in the UK and internationally, towards curriculum reform integrating epistemic insight (knowledge about knowledge, including about what disciplines are and how they interact), with significant implications for the teaching of science in schools. We present findings from two exploratory workshops with 15–17-year-old students in England on the role of science during the pandemic. We found that the workshops provided space for students to begin to develop epistemic insight regarding how science informs decision-making in dialogue with other disciplines. We make recommendations proposing pedagogical approaches using live, complex, real-world problems to address issues around understandings of the nature of science, misinformation, trust and participation in science.

## Introduction

Covid-19 has been described as the greatest challenge for the world since World War Two (BBC 2020a). At the same time, it is also incentivising international scientific research and encouraging teachers to be innovative and imaginative about what and how they teach (Zhu and Liu 2020). The importance of global issues for individuals and society, and their potential to stimulate student interest, makes them strong contenders for inclusion in curriculum and course design in schools and universities.

In this paper, we make a case for introducing pedagogical tools and assessment frameworks based on the Epistemic Insight curriculum framework, an approach that includes developing students’ understanding that different questions and problems call for different disciplinary approaches. We show that workshops we designed to develop epistemic insight can be used to strengthen and test students’ understanding of the nature of knowledge and their appreciation of the importance of multidisciplinary approaches. We do this by presenting findings from an exploratory, small-scale research project with students aged 15–17 from two English schools which sought to evaluate a workshop on Covid-19 and science, based on the Epistemic Insight curriculum framework. The workshop was designed to develop students’ understanding of the nature of science and the relationships between science and other disciplines in the context of real-world problems.

## Science and Covid-19

The Covid-19 pandemic has put science into the public spotlight. For the first few months of the pandemic, in the UK as elsewhere, government ministers frequently said that they were ‘following the science’ in their decisions to lock down businesses, recreation and family life (Scally et al. 2020). However, sometime later, the use of this rhetoric reduced alongside an increasing public awareness that science on its own cannot provide a simple and complete solution to complex real-world problems (Stevens 2020). For example, when the prime minister discussed the question of when to send children back to school, he described it as a matter of morality—saying that morally, adults have a duty to give children access to the education they deserve (BBC 2020a). A view on the significance of the role of science was addressed by the Deputy Chief Medical Officer for the UK Jonathan Van-Tam, who stated during a press conference that decisions about Covid-19 are a combination of ‘science, politics and practicality’ (quoted in Neilan 2020). Van-Tam’s comment indicates a more nuanced understanding of how science informs decision-making. There has since been more emphasis in ministerial speeches and in reporting of the pandemic of the necessity of a multidisciplinary understanding of the Covid-19 pandemic and of a collaborative, multidisciplinary approach to addressing the problems it raises (Moradian et al. 2020). Covid-19 has prompted examinations in the media and elsewhere of the relationships between science and other disciplines (Er Saw and Jiang 2020). Questions about how forms of knowledge, evidence and argument which arise from taking different disciplinary perspectives should be weighed up in decision-making are also of interest to educators and educational researchers (Fischoff 2020). For example and in particular, school science education has an important role to play in helping young people to develop their appreciation of where, why and how science informs our thinking about complex real-world problems (Reiss 2020).

Conducting their research before the pandemic, the UK ‘Public Attitudes to Science’ survey investigated the public’s sense of involvement and participation in science; the findings from the survey indicated that “although most people felt the public should play a role in science decision-making, there was relatively little desire for personal involvement: 28% expressed an interest in involvement, while 69% were happy to leave decision-making to others” (2019, p. 10). The report noted that having higher science capital (i.e. the extent to which an individual feels that science is ‘for me’) was highly correlated with positive views about science. Previous research has shown unequal distribution of science capital according to gender, ethnicity and cultural capital (Archer et al. 2015). Furthermore, research reveals that the pandemic is exacerbating a variety of educational inequalities (Andrew et al. 2020). It seems to us to follow that educators have a responsibility to help students to understand the role of science during the pandemic and in so doing should pay particular attention to ensuring that their approaches effectively engage students with lower science capital.

The proliferation of online misinformation is another central educational concern related to science and Covid-19. One BBC report (February 2020b) identified a ‘viral video’ in which anti-vaccination messages were presented by characters wearing medical garb filmed against a clinical backdrop. In this way, the video exploits visual clues that are associated in the public mind with expert knowledge and experts to trick viewers into trusting the message that was being promoted. It illustrates and adds to the case that science educators have an important role to play in raising student resilience to misinformation (Tseng 2018). Indeed, many are already recognising this responsibility—research with 6000 teachers conducted in England in August 2020 shows 75% of science teachers had talked or expected to talk to their students about Covid-19 misinformation (Billingsley et al., under review).

We can thus observe several important issues concerning the communication of science during the pandemic, including the need for multidisciplinary responses to Covid-19 and the threat of proliferating misinformation masquerading as science. We turn next to the question of the suitability of current approaches to science curriculum and teaching for meeting this need.

## Contexts of curriculum reform

There is a trend, both in the UK and internationally through initiatives prompted by the Organisation for Economic Co-operation and Development (OECD), towards curriculum specifications and strategies that seek to reduce knowledge fragmentation by emphasising that students will need multidisciplinary ways of reasoning to address real-world problems (Steiner and Scherr 2013; Stentoft 2017). For example, a concept note from the OECD’s *Future of Education and Skills* project states:

“Over the past few decades, there has been growing emphasis on thinking of the world as made up of inter-related systems, rather than solely as a series of discrete units. Education systems around the world have been moving from defining subjects and required curriculum knowledge as collections of facts, towards understanding disciplines as interrelated systems” (2019, p. 5).

We can see an example of this in the UK context by observing changes taking place within Ofsted (Office for Standards in Education, Children’s Services and Skills), a department of the UK government responsible for inspecting education and young people’s services. The commentary of teachers, school leaders and educational theorists around Ofsted’s new inspection framework (2019) has highlighted the emerging importance of schools teaching both substantive knowledge—knowledge content—and disciplinary knowledge—knowledge about how the discipline works. As Amanda Spielman, Ofsted’s Chief Inspector, argued, the curriculum should not be formed from “isolated chunks of knowledge, identified as necessary for passing a test. A rich web of knowledge is what provides the capacity for pupils to learn even more and develop their understanding” (Spielman 2018).

There is potential confusion in language between the OECD’s terminology and the language around the new Ofsted framework. The OECD’s term ‘disciplinary knowledge’ corresponds to the term ‘substantive knowledge’ used in discussions around the new Ofsted framework. As the *Future of Education and Skills* documentation puts it, “disciplinary knowledge includes subject-specific concepts and detailed content, such as that learned in the study of mathematics and language” (2019, p. 4). However, in this paper, we use the term ‘disciplinary knowledge’ as it is used in the academic literature and in the UK context. This has been defined, for example, by Kelly et al. as “developing identity and affiliation, critical epistemic stance and dispositions as learners participate in the discourse and actions of a collective social field” (2008, p. ix). The OECD refers to this as ‘epistemic knowledge’—their analogous definition is “the understanding of how expert practitioners of disciplines work and think” (2018, p. 4). Another relevant category used by the OECD is ‘interdisciplinary knowledge’, which they define as “relating the concepts and content of one discipline/subject to the concepts and content of other disciplines/subjects” (ibid.).

Epistemic insight has been defined as ‘knowledge about knowledge’ (Konnemann et al. 2018) and it facilitates access to all four of the OECD’s categorizations of knowledge, including both how disciplines work and how disciplines interact (Billingsley et al. 2018). This includes the ability to differentiate and point out similarities and differences between disciplines in terms of their preferred questions, methods and norms of thought, and be able to apply these insights in their learning. For example, students may work with a ‘bridging question’ like ‘Why did the Titanic sink?’, considering which disciplines can help us to answer this question and why. ‘Big Questions’ (i.e. questions that concern the nature of reality and/or human personhood) that require multiple disciplines to answer can be used to develop understanding of the natures of science, religion and the wider humanities (Billingsley et al. 2017). Epistemic insight has been used more widely as the basis of interventions with pre-service science teachers (Erduran and Kaya 2018), lessons on evolution and creation (Konneman et al. 2018; Owens et al. 2018), the promotion of STEM careers (Lawson et al. 2020) and teaching science to Australian indigenous students (Michie et al. 2018). In the context of this paper, it is the basis for an intervention to strengthen students’ capacities to think critically about the nature, application and communication of knowledge, especially scientific knowledge.

The curriculum in most countries, including all four UK home nations, includes both substantive and disciplinary knowledge. However, in England particularly, school science has become increasingly compartmentalised away from the bigger questions explored in the humanities, with teachers working through a checklist of scientific knowledge-bites that often come up in exams (Sibieta and Jerrim 2021, p. 37). Decades of research highlights that a tendency to only test substantive knowledge in science (i.e. facts and ideas produced by science) leaves many young people with a fragmented and often overly simplistic idea about how science works. The design of examinations, the structure of timetables and the lack of collaboration between teachers in secondary schools help to lock these practices in place and are barriers to change (Yager 2000). Entrenched compartmentalization—when the separation of subject classrooms becomes a habit that dictates students’ and teachers’ expectations about what should happen in the classroom—means that students do not see a cross-disciplinary question being addressed through multiple perspectives (Billingsley et al. 2017). With reference to our earlier discussion, Covid-19 has put the processes by which science informs policy and governmental decision-making front and centre in the media, highlighting the need for science to be considered together with other disciplinary perspectives. This draws renewed attention to the need for continuing reforms in how we talk about knowledge in the curriculum. It also raises wider questions, such as how to develop a broader and richer conception of knowledge and science fit for the integrated thinking required in a post-pandemic world.

This question is a central concern of the LASAR (Learning about Science and Religion) Research Centre, (where this research study is based). As we began to consider these questions about the public’s perceived role of science in the pandemic and its connections with science education, we began to consider whether there might be strong pedagogical potential for engaging young people with these issues. We then considered how we might design and evaluate educational interventions based on these ideas.

## Epistemic insight, science and real-world problems

One of the overarching goals of (LASAR) is to understand how knowledge is being engaged with in classrooms and how we can teach more effectively about the nature of knowledge (Billingsley et al. 2018). As we noted previously, one aspect of epistemic insight is understanding that knowledge tends to be organised into disciplines, each with preferred questions, methods and norms of thought. As such, the aim of developing students’ epistemic insight supports the aim of developing ‘disciplinary knowledge’ (in Ofsted’s terminology). However, it also builds on and enriches interdisciplinary knowledge (in the OECD’s terminology) by requiring that students are able to apply their disciplinary knowledge to explain and compare the strengths and weaknesses of different disciplines when making decisions about real-world problems.

Epistemic insight aligns closely with educational research agendas working towards interdisciplinary and experiential learning (Nikitina 2006; Morris 2020). Research has shown that an experiential learning approach (Kolb 2014) can enhance the teaching of school science (Cheng et al. 2019), findings that support the application of epistemic insight. Similarly, the interdisciplinary, problem-based pedagogy utilised in epistemically insightful approaches can be seen in recent research on the importance of including controversial socio-scientific issues in science education (Bayram-Jacobs et al. 2019). Epistemic insight also interacts with parallel developments in science education research concerned with the preparation of science teachers to work across disciplinary boundaries (Handtke and Bögeholz 2019) and how the epistemology of science is framed in science education (Papadouris and Constantinou 2017).

**Fig. 1:**
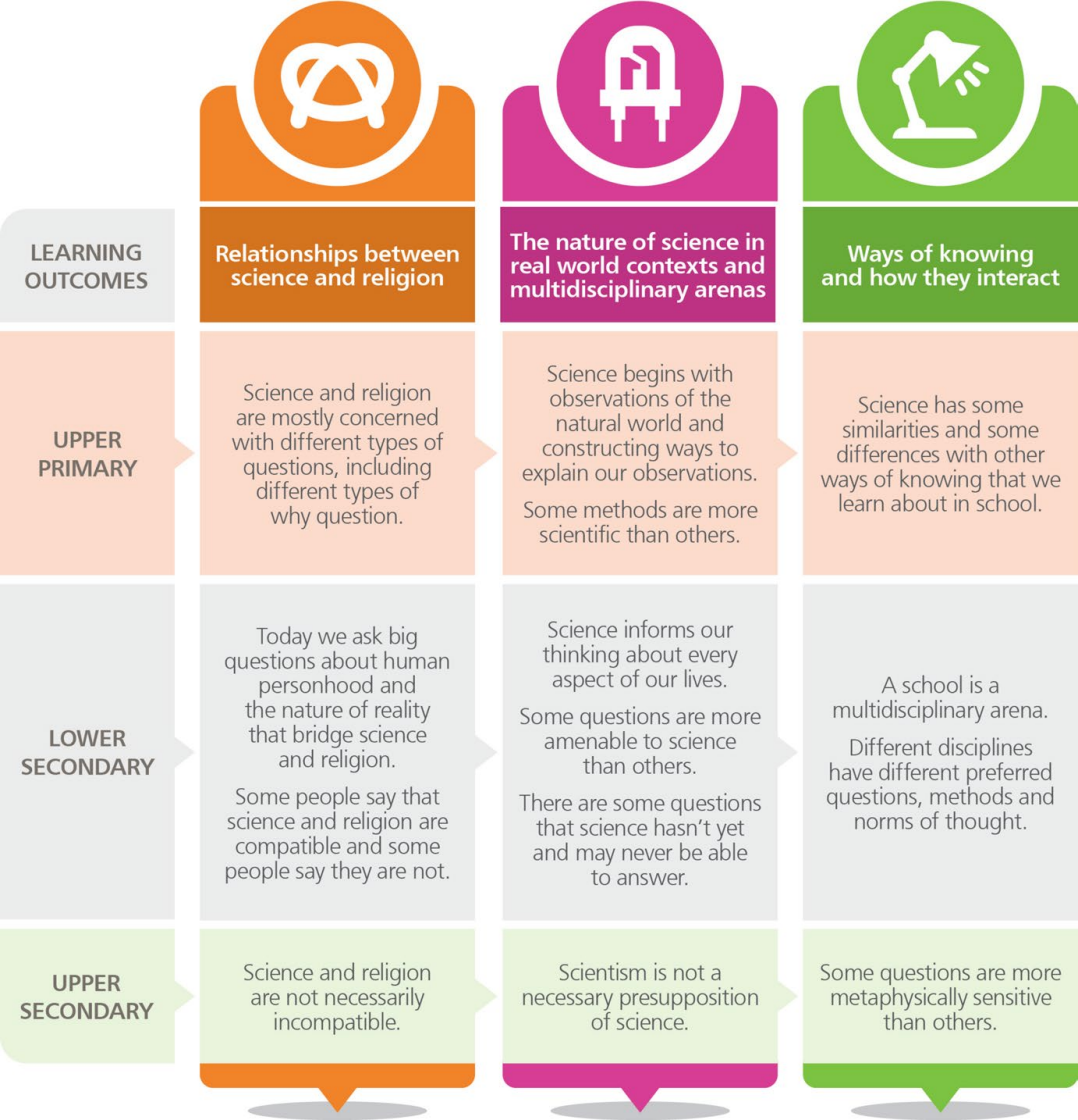
Epistemic insight curriculum framework (Billingsley et al. 2018)

Before the pandemic, we were developing interventions and resources working across disciplines and subject boundaries with the aim of helping students to access epistemic insight (e.g. Billingsley and Nassaji 2019a, 2019b). The increasing visibility of science in the media throughout the Covid-19 pandemic presented an opportunity to explore the potential of a discussion-based workshop engaging with Covid-19 and science. We drew on the Epistemic Insight curriculum framework (see Fig. 1) to design the discussion guide for the workshop, working towards specific learning objectives (discussed below).

Our aims in designing and testing this workshop were to (a) investigate the strengths and weaknesses of the workshop in helping students work towards learning objectives, (b) evaluate how students engaged with the discussion and (c) assess the potential of the workshop for developing students’ epistemic insight. The research was undertaken with a view to informing the design of more systematic programmes and assessments that could investigate the potential of engaging all manner of real-world problems for similar purposes. Therefore, we posed research question for this study:

Can workshops discussing science and Covid-19 develop students’ epistemic insight – particularly regarding the relationship between science and other disciplines?

## Methodology

Given the paucity of existing work investigating students’ learning of disciplinary science knowledge through real-world problems, an exploratory approach was best suited to the aims, objectives and research question posed (Lune and Berg 2017, p. 176). An exploratory approach also supported the intention of this study to be a test for feasibility which could then be built upon if successful. Our sampling approach was based on convenience, suitable for a small-scale, exploratory study (Maxwell 2012).

We designed a workshop and enlisted two teachers to run the workshop in 2 separate schools, with a total of 11 student participants. We made recordings of the workshops which functioned as our source of data for analysis. Utilising existing relationships with teachers who could facilitate the workshop with their current students ensured that the data would not be unduly effected by the process of establishing new relationships of trust that would have been necessary through a more direct, researcher-led approach. By enlisting teachers as facilitators, our study benefitted from the ‘prolonged engagement’ of teachers with their students (Erlandson et al. 1993, p. 133), one of the markers of credibility in a qualitative study (Shenton 2004).

Workshops are not a common methodological tool in educational research, though they have been used in a variety of settings, e.g. to assess critical thinking interventions (Ahmed and Asraf 2018). In their paper considering workshop methodology, Ørngreen and Levinsen (2017) note three ways of thinking about workshops (a) workshops as a means, (b) workshops as practice and (c) workshops as methodology. Our study aimed to both facilitate learning (workshops as means) and to generate data that would illuminate the interactive processes and progressions underlying the learning (workshops as methodology). In our study, the workshop was designed to elicit student’s engagement and understanding of the framing of science during the pandemic and to provide space for students to discuss various ways that science has been framed during the pandemic, including through the media and through government policy. Questions were designed deliberately to allow teachers to support students in working towards the Epistemic Insight learning objectives in such a way that students learning processes might be observed. The workshop used in our study is thus different from, for example, a focus group, insofar as there is an intentional effort to facilitate learning alongside the elicitation of data.

We chose three relevant learning objectives in the Epistemic Insight curriculum framework that were most relevant to the aims of our study. This included three learning objectives under the heading “The nature of science in real world contexts and multidisciplinary arenas”:

1. Science informs our thinking about every aspect of our lives.
2. Some questions are more amenable to science than others.
3. There are some questions that science hasn’t yet and may never be able to answer.
  This also included one learning objective under the heading “Ways of knowing and how they interact”.
4. Different disciplines have different preferred questions, methods and norms of thought.

We then designed a series of discussion questions designed to facilitate these learning objectives. Below is the schedule of questions for the workshop:

- Do you/did you follow the news on Covid-19?
- We have frequently heard the terms such as ‘we are led by science’, ‘we are guided by science’ from the government ministers? What does this term mean to you?
- In one of the daily Covid-19 updates, the Deputy Chief Medical Officer for the UK, Professor Jonathan Van-Tam, said that decisions that are made are ‘always a complex blend of science, politics and practicality’. What do you think about this comment? Is this one different from the phrase ‘we are led by science’?
- What do you think they mean by ‘science’?
- What if science cannot answer a specific related to Covid-19? Can you think of an example?

A further 5 questions had been designed providing further opportunities to work towards learning objectives; however, these were not reached in either session due to students not progressing as quickly as anticipated.

Each question was designed to contribute to one of the three identified learning objectives of the Epistemic Insight curriculum framework, as follows:

- Question 1 was designed to get students ‘warmed up’ to the subject by allowing them to talk about their own experience of engaging with the media during the pandemic.
- Questions 2 and 3 were intended to open the discussion and help students to notice and achieve learning objective 1, that “science informs our thinking about every aspect of our lives”—in this case making explicit how central science has been to informing thinking about Covid-19.
- Questions 3 and 4 engaged closely with learning objective 3, “different disciplines have different questions, methods and norms of thought”.
- Question 5 engaged explicitly with the limits of science in informing thinking about Covid-19 with the purpose of engaging students with learning objective 3 “There are some questions that science hasn’t yet and may never be able to answer”.

Due to the limitations of time resources and school availability during a pandemic and in light of the exploratory nature of the study, schools were recruited using personal contacts of the research team. Informed consent was obtained from all participants and institutional ethical procedures followed at all stages. Recordings from both workshops were transcribed and anonymised, identifying voices of the discussion participants across the different questions. All participants were 15–17 years old. Below is a table of information about the workshops and their participants (names have been swapped for pseudonyms):

## School and participant information

**Table:** 

Workshop	Facilitator	School type	Participants (Age)
1	Lana	High-performing suburban secondary school	Nida (17) Rachel (15) Sana (17)
		High-performing suburban secondary school	Aisha (15) Ellie (15) (Present, but did not contribute to discussion)
2	Sean	Urban private secondary school	Beatrice (15) Matilda (16) Olivia (15) Verity (16) Lauren (16) Emily (15)

Recordings were transcribed by a member of the research team. A second researcher checked the transcription and added punctuation for clarification, e.g. question marks where students expressed uncertainty. The data were analysed using a thematic approach (Braun and Clarke 2006), initially by one member of the research team. We began by reading through each transcript to familiarise ourselves with the data. In our first round of theme generation, we treated each question as a distinct ‘unit’, developing themes within and across the 2 transcripts within each question. These were then condensed into a single theme denoting primary finding for that question. This formed the structure for how we report the data below, proceeding chronologically through the workshop. These themes were checked and refined by a second member of the research team. In our second round of theme generation, we treated each transcript as a whole ‘unit’, generating themes focussed on how students were developing and changing their responses as the discussion unfolded. These themes were integrated into the reporting of the data within the existing structure. In this way, we were able to integrate our within-question findings with the broader patterns of change across the discussion.

## Findings and discussion

### Considering how science informs decision-making about Covid-19

Questions 2 and 3 were designed to work together to progress students from the more simplistic idea of the government being ‘led by the science’ to the more sophisticated idea of there being a ‘blend’ of science with other forms of knowledge. The latter, more sophisticated position, represents one of the insights we are interested in students developing, namely, an understanding of the role of science in informing decision-making through dialogue with other disciplines. We found that the students engaged positively with this part of the workshop. There were a variety of extended responses from a range of participants. Further, we can see evidence of the different aspects of epistemic insight we hoped the workshop would develop.

When responding to question 2, students initially attributed a range of meanings to being ‘led by science’ that demonstrate significant conceptual gaps in their understanding of the differences between science as a school subject and science used in real-world contexts. Sana responded first to the question, stating:

I mean, when someone says science you think of an organisation where scientists are involved? So not just the government ordering and saying, Okay, now you can go out in bubbles. And they're involved with the World Health Organisation and health scientists working on to see what's best for the population. So, I just think if you're led by science, there are scientists and people who follow science religiously. And they're trying to make decisions on what the population can do.

We can see here that Sana has begun to consider how science might inform decision-making, imagining the ‘conversation’ between the government and health scientists. This is an important step towards the learning objective “science informs thinking about every aspect of our lives” as Sana imagines how health science shaped the decision, e.g. to introduce ‘bubbles’ (a group of people with whom close physical contact is allowed under Covid-19 restrictions). Sana comments on the idea of the government being ‘led by the science’:

Mainly it’s the hospitals and all the workers in the hospitals that are working to treat people with Covid-19. And they’re still trying to find a vaccination for it… just kind of them supporting it. Oh, I don't know how to say this. But they have to... Healthcare has to work through it first and then maybe later on science can come into play because I don't think... science is quite slow. I don't think you can always rely on it.

Sana is having some difficulty in expressing her ideas but with the scaffolding of the focus group questions, we can see is moving towards an understanding that there are some problems such as vaccine development that are more amenable to science; there are some others such as how to care for sick patients that are less so.

Here, Sana is beginning to evidence the learning objective “some questions are more amenable to science than others” as she notices that science is a rigorous and time-consuming process of knowledge creation in contrast to the imminent demands of healthcare settings. This is an important progression in developing epistemic insight regarding the limitations of science in informing decision-making. A next step for Sana would be to recognise that healthcare is a multidisciplinary context in which many questions and problems are amenable to scientific investigation. Further, Sana has not yet recognised that the limitation of science in the quick decision-making of healthcare settings does not undermine its ‘reliability’ – rather it highlights its distinctive role and nature when addressing certain kinds of questions and problems.

The responses to question 2 in the second group were significantly different. Verity responded first, observing that the claim of being ‘led by the science’ reveals something about the government’s desire to present itself in a favourable light:

That the decisions they're making shouldn't be from a political stand? Like if they're going to open the lockdown it shouldn't be because they want to help the economy? It's because they're caring about our health?

This response led others to speculate on the underlying intentions of the phrase ‘led by science’. Lauren spoke next:

I suppose it's their way of suggesting they're making informed decisions. And it's kind of, it's kind of a cover of your back kind of thing to have to say, because it's just that you are following the suggestions that the scientists studying the virus are making. They have to suggest that yes, they have the public's health in mind, their best interest.

This discussion represents a step towards the learning objective “science informs our thinking about every aspect of our lives”. Verity and Lauren are recognising that science may take on a rhetorical function in society alongside the function it plays in informing decisions. However, the implicit suspicion and cynicism about government agendas (however justified) may be limiting the possibility of engaging with how science is *actually* informing the government’s decision-making. For this epistemic insight learning objective to be achieved, the use of science as rhetorical placeholder for authority needs to be considered *as well as* the actual processes of science informing thinking and decisions.

### Developing more nuanced understandings of how science informs decision-making

Group 2 demonstrated progression as they considered the new statement by Jonathan Van-Tam in question 3. Matilda immediately recognises the critical difference between the two statements:

Well, it's different than we are led by science, because this time, they're acknowledging that a lot of the decisions do obviously come from worrying about politics or the economy. Which we've seen a lot of the time, a whole big thing with the government these days is has been restarting the economy. And so this phrase is showing that a lot of their decisions, whilst sometimes influenced by science are also based off of that.

Subsequent responses from Lauren, Olivia and Emily in group 2 all restate and rearticulate this basic observation about the greater nuance present in Van-Tam’s statement. There is also a clear value judgement being made that Van-Tam’s statement better represents the role of science—it is described by Lauren as more ‘balanced’ and by Emily as more ‘realistic’. We can see that students are beginning to grasp the epistemic insight being displayed by Van-Tam—the need for recognising multi-disciplinarity. However, at this point, the discussion with group 2 has not progressed to how these ‘other concerns’ might interact with scientific evidence. This is the level of epistemic insight that we would expect from students aged 15–17 within the Epistemic Insight curriculum framework. Further, Emily’s final statement after reflecting on Matilda and Lauren’s is worth noting:

We have to kind of find a midpoint and find a way to bring everything together. And yes, I think that's in a sense why the medical officer is saying this. It's not just as simple as saying we need to be kind of guided by science, we in reality, we need to be guided by lots of things, obviously, primarily by science.

We can also see this in the first response given by Sana (group 1) in relation to question 3:

I mean, it's very different to saying we are led by science. Because when you say you're led by science it leads the viewers and the people who are listening in to think that its only science involved. But when it says now that the medical officer said that there’s ‘science, politics and practicality’, this also takes into account other people's views that that might not be so involved in science. That makes the opinion and the information given out a little less reliable because they're trying to think of how to say it. They're not just trying to focus on the population and the health of everyone, they're also trying to think, how will this affect the future? How is that going to affect politics? So I think it just makes it that little bit less reliable than if we're just led by science.

For Sana, science itself is compromised when combined with other practical and economic concerns—the considerations that come when applying science to a real-world problem and the need to consider a variety of contextual factors make science less reliable. Here, Sana is grasping an important point relevant to the learning objective “some questions are more amenable to science than others”. Sana’s comment suggests she has an understanding that science has distinctive characteristics compared with many other disciplines and in particular disciplines in the humanities. This insight helps to explain why real-world problems, like those created by a pandemic, cannot be ‘solved’ using science alone. However, at this point, Sana assumes that a multidisciplinary approach in which science is supplemented by other disciplines is problematic, rather than as reflecting something inescapable about the nature of our complex world and the different forms of knowledge we need.

Nevertheless, Sana follows up her comment with a modest correction of her position which shows clearly how the workshop is providing space for progressing students’ epistemic insight. Sara (the teacher) responds to Sana’s previous comment:

Sara

And, you know, when you said other factors apart from science, so you're suggesting, like politics, or economics, or other things, psychology or whatever, they're not science.

Sana

It's not that they're not science. It's just when you think of science, you think of people higher up just focussing on the virus and how to overcome it, rather than also thinking of the economy or how it affects... I guess it is part of science, how people are affected by it. So the psychology of people who get it or family members who have it. But I think that we should just be focussing on science as a whole, also taking into account the economy and people's well-being. But it doesn't exactly tell us about the blend, how the blend is split? I don't really know what to say but I think it's a good blend, because it allows everything to be taken into account for that may be focussing more on the science, because now that they're saying numbers have gone down, or people are recovering, maybe instead of focussing on social well-being and economy, and the NHS, we should focus on treating them focussing more on the science side of it, and actually figuring out what it is.

Here, Sana may be struggling to define the border around science: if we study the impact of the economy on people’s wellbeing or if we look at the psychological impact of the pandemic on family then are these ‘scientific questions’. Sana begins to visualise the complex relationships within the problem—and notes that Van-Tam’s comment does not answer is ‘how the blend is split’. Nida continues the discussion, affirming Sana’s point and restating the idea of science needing to be contextualised, summarising “it’s just a matter of having to look at the situation”. This idea of contextual variation is again an important element of insight about the nature of science and its relationship with other disciplines. However, development of this aspect of epistemic insight requires opportunities to consider specific examples of *how* context might dictate which forms of knowledge take priority. Rachel refers to the teacher’s earlier paraphrase that “other factors apart from science, so you're suggesting, like politics, or economics, or other things, psychology or whatever, they're not science” and agrees adding:

Rachel

But I think that science should still remain the priority. So if it's a complex mixture of all three of them, science should always remain the most important one, because the health of the population is what's most important.

Sara (the teacher)

So... do you think that in government should look into numbers, and medical side of this story is finding out about the virus?

Rachel

Well, I think that's the most important but the government should still take into factors such as the economy, practicality and everything else.

Whilst we can observe some progression as students notice the difference in nuance between question 2 and questions 3, there are still conceptual gaps and misperceptions that limit the epistemic insight being advanced. Rachel gives voice to two ideas that students are not yet able to consider at the same time—that science needs other perspectives to supplement it, but that science should *always* have priority, or else it risks losing its reliability as it is ‘blended’ with other disciplines. Whilst Sana does introduce the idea that the blend of science with other disciplines in context needs considering, the idea that science remains unquestionably top of an imagined hierarchy of authority applicable to all situations remains.

### Widening what might count as ‘science’ in responding to Covid-19

Question 4 asked participants “What do you think they mean by science?” (referring to the statements in question 2 and 3). The intention here was to allow students the opportunity to develop the discussion further by considering the differences between the two statements in question 2 and 3, focussing on the scope and meaning of ‘science’ in different contexts. The intent here was to observe processes that would help students notice that science, as it is used in public settings, is considerably more complex than science as it is presented in educational curricula.

In group 2, the question about the meaning of science did not seem particularly effective. Students did not grasp the intention behind the progression of the questions towards considering the differing meanings of ‘science’ across the two statements. Instead, they resorted to more abstract definitions of science that did not advance the development of epistemic insight, i.e. the improved understanding of how science work in real-world contexts that Jonathan Van-Tam’s statement represents. Group 1’s discussion in response to this question began in much the same way with vague, short statements about science as “theories, facts and statistics”. However, an interaction between Sara (the teacher) and Nida saw a new understanding emerge:

Nida

I'm not sure. Because, I think science can be all that way. It's also... psychology, is kind of like a science. I mean, when we talk about being led by science or stuff like that, we think of sciences like biology, and they said some sciences, but psychology is also science, and it's a social science, but that is also an important thing to take into account. So, I think it is just also analysing, I don’t know, people's mental health, and that kind of stuff, too.

Sara

Well done. Yeah. True. So, are you suggesting that science is wider than both biology and chemistry?

Nida

Yeah, I feel like when they talk about it on the news, the first thing that comes to mind is biology and chemistry, but it's much wider and includes a lot more subjects.

Sara

Well, when you heard it at the beginning, were you thinking the same way as you think now, or some conclusion that you came up to? Now, after a while?

Nida

The thing is, just like I said, when you first see it, biology and chemistry come to mind. So, when you first said that, that's what I was kind of thinking about, but as I thought about it a bit more than I was like yeah psychology and others are also included in science. So yeah, I kind of got to this conclusion after the questions.

Here, Nida has begun to move to a more sophisticated and critical understanding of science in relation to the learning objective “different methods have different questions, methods and norms of thought”. The idea of science as constrained to school subjects is clearly present in Nida’s previous discussions of science as being either about understanding the nature of the virus itself and how to prevent transmission. The idea of scientific expertise needing to be ‘contextualised’ for the real world is also now becoming more solidified and anchored in the language of disciplinary differences. She has also begun to gain epistemic insight around the learning objective “different disciplines have different questions, methods and norms of thought”. Discussing the relationship between the discipline of science and psychology would have advanced their engagement with this learning objective, providing further opportunities for students to examine differences between disciplines and how scientific thinking works in disciplines outside the natural sciences.

### Noticing the limits of science in responding to Covid-19

Question 5 asked “What if science cannot answer a specific question related to Covid-19? Can you think of an example?”. Although we may have expected students to be less prepared to talk about the limits of science given their emphasis on science’s hierarchical superiority as a discipline, both groups engaged well with the idea that science may not be able to answer all the challenges of Covid-19. In group 2, Verity expressed the following, with some hesitancy:

I don't know if this is linked to the question. But… there's the question of if you can force people to stay at home, and yet… and yes, science does say that you're safer staying at home? But it can't really decide on the ethics behind that.

Verity has immediately noticed that science is unable to give a decisive answer on what we *should* do in response to the evidence it provides about virus transmission and noticed that this is a question for ethics. Paul (the teacher) helpfully draws out Verity’s point, making an important observation drawing a distinction between the evidence that science provides about vectors of transmission and the ethics of enforcing restrictions:

The scientists might say, if you're going to sneeze and you haven't got a tissue, do it into your elbow, but you sort of making people do that isn't quite the same as sort of saying, science says, if you don't do this, it's going to spread very easily.

This is a valuable contribution—Paul is helping the students notice the relationship between disciplines and that the contributions of science and ethics are distinctive and deserving of separate consideration. The discussion has progressed towards the learning objective “some questions are more amenable to science than others”. However, the conversation did not progress any further. There may have been an opportunity here to develop Verity’s epistemic insight further, e.g. by thinking of other cases in the problem of Covid-19 where ethics is required to interpret and apply the evidence supplied by science. This could have been done by considering precisely why questions about virus transmission are amenable to science but not questions about how we should act in response.

In group 1, the discussion was longer and eventually moved away from considering the limits of science to considering other negative and positive side effects. In the course of this discussion however, Nida makes an important observation:

Um, I just feel like when I heard about the Covid-19, I was just really confused about why it doesn't follow the normal rules that we learned about viruses because I don't know it lasts much longer on surfaces because I've heard so many examples of how someone was and sitting somewhere and let's say they had the Covid-19 and they left and then I don't know a day later someone else came and sat there managed to get the Covid-19 or, for example why in some of the warmer countries, the viruses, the number of people that are infected is a lot higher. So because you think that in warmer temperatures, viruses wouldn't survive, but they are surviving. So it's just a bit confusing, how do we explain that?

It is notable here that Nida has conflated a question which science is *unable* to answer with a question that is amenable to science but has not yet been answered (i.e. the apparently unpredictable behaviour of the virus across different geographic contexts). Further discussion could have deepened engagement with the learning objective “There are some questions that science hasn’t yet and may never be able to answer” by explaining that although some ‘frontier’ science may be in a state of flux as to how it is engaging with evidence, it is within the scope of scientific method to at some point have a clearer empirical answer to questions about the behaviour of the virus.

The above discussion of our findings has highlighted a consistent pattern that gives a clear answer to our research question. Students consistently displayed the beginnings or first steps of the three epistemic insight learning objectives that we built into the design of the workshop. However, in all instances, the epistemic insight was incomplete, fragmentary, or under-articulated. Despite this, in several instances noted above, students self-corrected, expanded on, revised, and reflected on their own thinking about the nature of knowledge and took advantage of the topic discussion to advance towards greater epistemic insight. Contributions both from the teachers and as a result of students building upon and responding to each other’s ideas provided opportunities for several participants to clearly progress in their understanding of the nature of knowledge drawing on discussions about Covid-19 and science. It should be noted that a limitation of this research is that we did not design the workshop to allow every participant the opportunity to display or reflect on the progression in their epistemic insight. The design of the workshop did not allow for us to assess the evenness of development in epistemic insight—some members of the group may have advanced more than others. Future research should include more formalised elements of assessment and feedback to address this (discussed below).

## Recommendations

In our introduction, we explained that the intervention was conceived as a way to increase students’ epistemic insight—or in other words, to strengthen their capacities to think critically about the nature, application and communication of knowledge, especially scientific knowledge. We explored a range of justifications for developing and examining students’ epistemic insight in the classroom. These include building appropriate trust in science and scientists in their contribution to real-world problems; encouraging students to be curious about nature, power and limitations of science in real-world contexts, having a sense of involvement and participation in science as a collective human endeavour, and enhancing students’ capacities to detect where claims are unscientific or science is being misinterpreted or misrepresented in media sources. The four recommendations below connect the findings of the workshops to these justifications, discussing the potential for future educational strategies and tools that use real-world problems to develop students’ epistemic insight, particularly their capacity to think critically about the nature, application and communication of science. We comment how epistemic insight might contribute to addressing public trust and participation in science along with the capacity to detect where unscientific claims are made or science is misrepresented to spread fear or challenge a policy or decision.

### (1) Engage students with how science works on ‘frontier’ questions and challenges

At the start of the workshop, Sana noticed that the practice of caring for the sick presents the need for an immediate response that seemed to her out of alignment with the apparent ‘slowness’ of science. Similarly, Nida observed that science didn’t seem able to account for the apparent discrepancies in the behaviour of the virus in comparison to other viruses and across geographical contexts. Much of the science informing the UK government’s decisions about Covid-19 has been ‘frontier science’—in some cases, the first studies exploring the behaviour of a new and unknown virus have been very influential in shaping policy and decision-making. The nature of frontier science is substantially different, for example, from the established theoretical body of scientific knowledge about how vaccinations work. Therefore, when the frontier science of Covid-19 shifts, it can be perceived by the public as undermining the authority of science, or, as Nida said, of highlighting its ‘slowness’ as being behind the curve of what is imminently required to act. It is therefore important for students to access conversations about how science shapes decision-making. This will prepare them to navigate the range of ‘scientific’ misinformation proliferating online by helping them notice that the openness of science to change and the ability to account for new evidence is in fact one of its strengths in informing responses to changing situations. This is part of a growing agenda within the education research community, with a recent report from the UK government’s Department of Culture, Media and Sport (DCMS) arguing that digital literacy should be the ‘fourth pillar’ of education against the growth of online misinformation (Digital, Culture, Media and Sport Committee 2018). Following this, (Anonymized) are currently publishing research and developing resources addressing misinformation through fostering critical thinking about the nature and application of scientific knowledge, helping young people engage effectively with science in its real-world decision-shaping capacity.

### (2) Provide a critical lens on how science informs decision-making in multi-disciplinary contexts

Group 2’s discussion of the language the government was using around science seemed to show a suspicion that ‘science’ was being used as a rhetorical placeholder to sustain public support and approval. As noted above, the workshop aimed and to an extent, succeeded in building students’ capacities to appreciate the nature of science in real-world contexts and thence to critique the motivations and nature of science communication during the pandemic. However, further development is needed to help students towards a nuanced understanding that includes political considerations alongside other disciplinary perspectives. This is a vital part of students developing a sense of ownership and participation in science as a collective human endeavour.

Previous research has argued for the effectiveness of teaching in an epistemically insightful way about complex real-world problems for engaging students with low science capital (Lawson et al. 2020). (Anonymized) has several forthcoming resources that address this, including a role-play of a decision-making committee who must take into account multiple perspectives to inform their response to a complex real-world problem. The intention of this exercise is not for them to make the ‘right decision’ but to notice and develop the epistemic insight that is required in those situations. However, concerns around the politicisation of science that students noted in the sessions also ought to be followed up (for example, through team teaching across politics and science classrooms) to develop richer understandings of the nuance and interaction in these real-world contexts. The substantial body of work on the role of socio-scientific issues in science education has already established a strong case for these kinds of interventions (Tsai 2018; Ke et al. 2020). Additionally, (Anonymized) is currently developing resources supporting teacher training in UK universities that supplement this work through a targeted focus on the connections between disciplines.

### (3) Explore how the nature of the problem being addressed shapes which disciplines are amenable to addressing it

Questions 2 and 3 were effective in setting up a simplistic understanding of ‘following the science’, introducing complexity (the ‘blend’) and allowing students space to notice the difference between the two. In the workshops, students engaged well with this approach and began to show epistemic insight as they noticed how practical limitations affect the way science can be applied to a problem. Members of group 1 grasped this idea, noting that how science is applied to a problem like Covid-19 depends on the surrounding contextual factors. Further development of this understanding would require examples and case studies to illustrate how context shapes the amenability of a question or problem to science. For example, the discussion could have progressed to consider that some questions about vaccine development and testing are amenable to science because they can be addressed by predicting and assessing the behaviour of the virus. Other questions that can be discussed to provide a comparison are how to transport vaccines to where they are needed, who to prioritise when administering vaccines and whether and to what extent vaccines should be sent overseas. The Epistemic Insight curriculum framework provides a progressive basis for building this understanding, aligned with the National Curriculum in science and its requirement that students understand “the power and limitations of science” (Department of Education 2014). Several studies on the impact of socio-scientific issue-based interventions have shown improvements in students’ understanding of the nature of science (Khishfe 2014; Khishfe et al. 2017). Building on this evidence, resources based on the Epistemic Insight curriculum framework have been developed to help teachers engage with real-world problems that are of imminent concern to young people in a way that helps draw out these crucial understandings of the nature of science in relation to other disciplines.

### (4) Teach about the limits of the science to combat misinformation

Discussions about the limitations of science were present throughout the workshop, despite not getting explicit attention. Students noticed that science was not well-equipped to inform questions about how to care effectively for sick patients ‘in the moment’ and that it could not ultimately provide an ethical framework to help you decide on how to restrict yourself. Nida’s discussion of psychology also raised questions about the boundaries of what could or could not be considered ‘scientific’. The discussion here could have proceeded—if there are aspects of psychology that are not ‘scientific’, why are they not scientific, and does this matter? Epistemic insight centralises the question of ‘what makes science, science’ (this questioning process is also applicable to any discipline). Part of this will involve helping students to appreciate that one of the features that distinguishes ‘scholarly’ work in science from pseudoscientific misinformation is the acknowledgement of a narrowly focussed research question and the recognition of current limits on knowledge. In developing epistemic insight into ‘what makes science, science’, students increase their capacity to evaluate the information they are receiving.

Epistemic insight thus has a key role to play in helping students sort out misinformation about live issues of global and national concern. This includes Covid-19 but could also apply to climate change, mental health, social injustices and new technologies. Misinformation is particularly damaging when it comes to issues like Covid-19 vaccinations, where a well-informed public is vital for a positive outcome for the wider community. Educational research drawing on scholarship about the relationships between religion and science has already explored the importance and potential of teaching about the limitations of science (Sollereder 2019; Bryant 2019). In this vein, resources and training for teachers, such as those being developed by (anonymized), will be essential for preventing the empirical authority of science being undermined and reducing the impact of the spread of potentially harmful misinformation about important issues.

## Conclusion

Covid-19 has changed the way that we think about science, particularly our understanding of how science works together with other disciplinary perspectives. This new awareness aligns with ongoing curriculum reforms, both at international levels through the OECD’s ‘2030’ programme, and nationally in England through Ofsted’s new inspection framework and the accompanying commentary highlighting the growing importance of disciplinary knowledge and the capacity to work across disciplines. The aims of (anonymized) and its progressive framework for developing epistemic insight speak clearly to these reforms.

This exploratory study with two groups tested the effectiveness of a workshop on Covid-19 and science for advancing students’ epistemic insight. We found that basing the workshop on the use of science in a real-world problem made a specific contribution to developing epistemic insight (students’ capacities to think critically about the nature, application and communication of knowledge) by engaging students with something highly relevant to their current lives and requiring them to think about real-world decision-making processes. However, we also noticed that students’ epistemic insight was patchy and underdeveloped, and opportunities to strengthen understanding were identified via the study. Our future research will work on refining the workshop structure proposed here, including CPD that will help teachers to guide the discussions effectively and methods of evaluation which will enable us to assess the effectiveness of the workshops in more detail.

## Data Availability

The datasets generated during and/or analysed during the current study are not publicly available due to ethical guidelines concerning anonymity of participants. However, they are available from the corresponding author on reasonable request.
